# Pulmonary Embolism Following Preterm Vaginal Delivery: A Case Report

**DOI:** 10.7759/cureus.71918

**Published:** 2024-10-20

**Authors:** Elham A Akbari, Komal Hazari, Deemah K Harb, Widad Abdelkareem

**Affiliations:** 1 Obstetrics and Gynecology, Latifa Hospital, Dubai Health, Dubai, ARE; 2 Internal Medicine, Latifa Hospital, Dubai Health, Dubai, ARE

**Keywords:** cervical incompetence, deep venous thrombosis, postpartum chest pain, preterm labour, pulmonary embolism, venous thromboembolism

## Abstract

Pulmonary embolism (PE) is a critical medical condition characterized by the obstruction of pulmonary arteries due to blood clots. The incidence of PE is heightened during pregnancy, primarily due to physiological changes such as altered blood flow and a hypercoagulable state.

This case report details a pulmonary embolism diagnosed immediately following a preterm delivery, despite the patient receiving prophylactic treatment with low molecular weight heparin (LMWH). The prompt initiation of therapeutic dosing of LMWH was vital in mitigating potentially fatal outcomes.

Accurate and timely diagnosis, coupled with appropriate intervention, is essential in preventing severe complications, including maternal mortality. It is imperative to implement effective risk assessment and prophylactic strategies to manage the risk of PE in pregnant women.

## Introduction

Venous thrombosis, a life-threatening condition, was first described in 1878 by Angus MacDonald following postmortem examinations of women who died during pregnancy or the postpartum period. The understanding of thromboembolism’s pathophysiology in these periods has expanded since 1856 when Virchow identified the triad of hypercoagulability, vessel wall injury, and altered blood flow as promoters of thrombus formation [1,2].

Pregnancy and the postpartum period are characterized by a hypercoagulable state, increasing the risk of venous thromboembolism (VTE) up to fivefold during pregnancy and 15-35-fold postpartum compared to non-pregnant women [1]. According to a WHO systematic analysis, pulmonary embolism accounts for 3.2% of global maternal deaths [3]. The CDC also lists pulmonary embolism as a leading cause of maternal mortality in the US, with cesarean delivery nearly doubling the risk of VTE [4].

Understanding risk factors and stratification is crucial for improving survival rates [4]. Treatment and management of pulmonary embolism depend on disease severity. New oral anticoagulants are effective and safe alternatives to traditional regimens, and recent trials do not support the use of cava filters in patients who can receive anticoagulants [5]. Thrombolysis significantly improves maternal survival rates in cases of massive pulmonary embolism [6]. In the UK, mortality rates have significantly declined following the mandatory implementation of antenatal and postnatal VTE risk assessment tools in 2010 [7].

This case report will describe the clinical presentation, including laboratory and imaging findings, management, and the course of illness in a patient diagnosed with bilateral pulmonary embolism following a preterm vaginal delivery.

## Case presentation

A 34-year-old gravida 4, para 3, with a history of a previous cesarean section at 9 cm dilation due to acute fetal distress, presented to the obstetrics emergency department in established preterm labor at 30 weeks gestation. During her current pregnancy, the patient was diagnosed with cervical incompetence and was admitted at 26 weeks for safe confinement. However, she discharged herself against medical advice. Following her discharge, the patient undertook long car journeys (approximately two hours) to reach her workplace while maintaining limited mobility. At home, she adhered to complete bed rest.

Using the Royal College of Obstetricians and Gynecologists (RCOG) VTE risk assessment tool, the patient was categorized as intermediate risk and was started on thromboprophylaxis at 28 weeks of gestation. She had no other significant medical history.

Upon admission, she received epidural analgesia and subsequently progressed to deliver vaginally within six hours. Approximately one hour post-delivery, the patient experienced acute chest pain, shortness of breath, and three episodes of vomiting.

On examination, she appeared generally pale. Vitals showed tachycardia (115 bpm), tachypnea (22 br/min), and desaturation with SpO2 reaching 88%. However, physical examination, including the assessment of the lower limbs, was unremarkable. As initial management, she was administered 2 liters of oxygen via nasal cannula, resulting in an improvement in oxygen saturation levels to 97-99%. Yet, due to high suspicion of pulmonary embolism, the patient was transferred to the ICU and initiated on a therapeutic dose of enoxaparin. 

**Figure 1:**
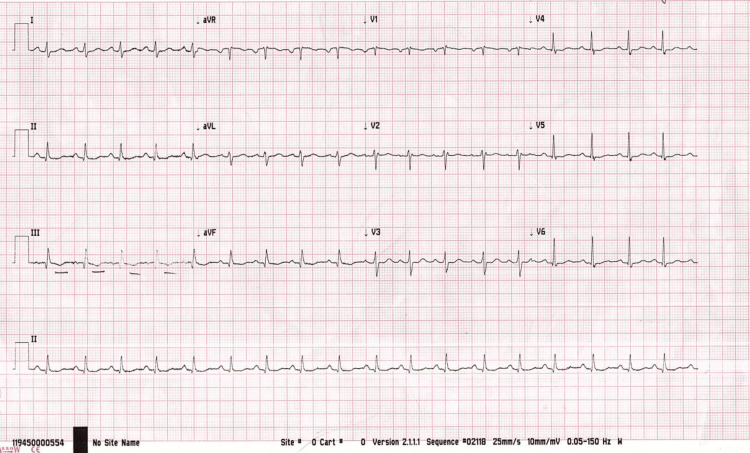
Electrocardiogram (ECG) Sinus tachycardia with T-wave inversion in lead III

**Figure 2:**
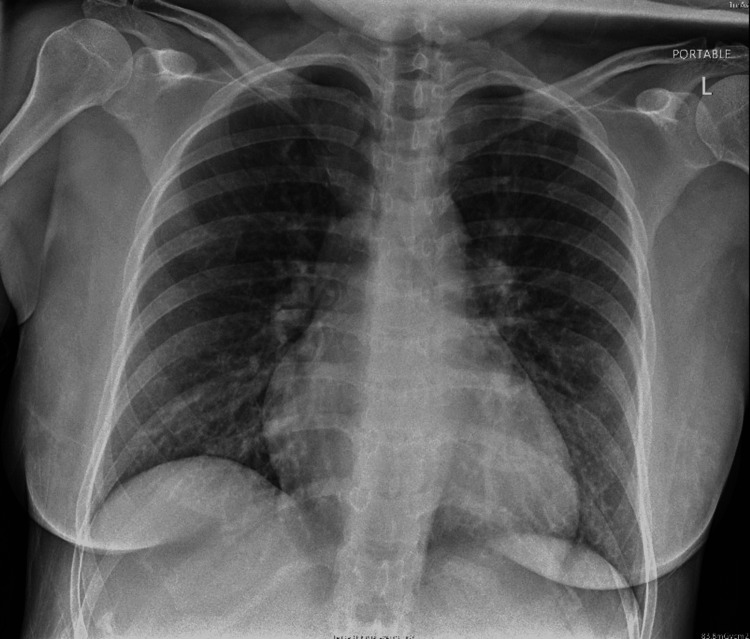
Chest X-ray (AP view) Chest X-ray showing prominent bilateral hilar broncho-vascular marking AP: anterior posterior

**Figure 3:**
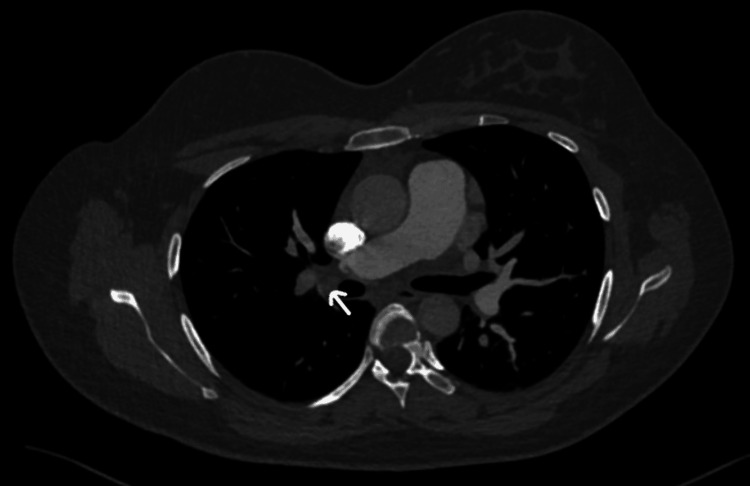
CT pulmonary angiogram (right pulmonary artery) There is evidence of filling defects seen in the right pulmonary artery

**Figure 4:**
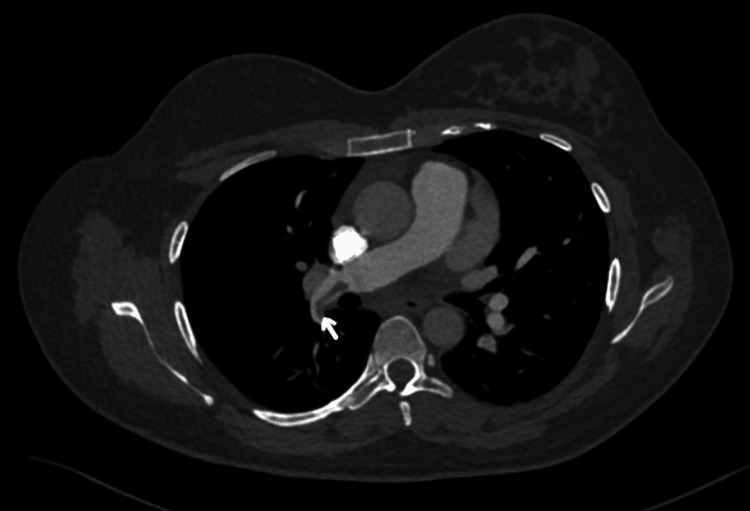
CT pulmonary angiogram (segmental branches of the right lung) There is evidence of a filling defect seen in the segmental branches of the right lung

**Figure 5:**
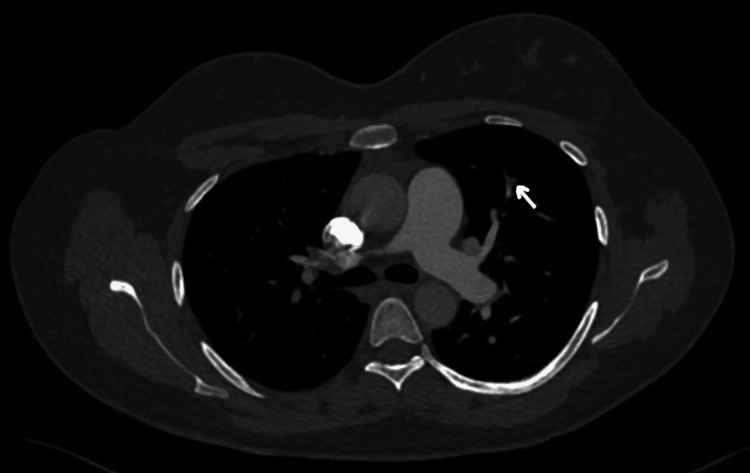
CT pulmonary angiogram (left segmental branches) There is evidence of filling defects seen in the segmental branches of the left lung

Initial investigations are presented in Table 1 with comparisons of repeated investigations on day four of starting low molecular weight heparin (LMWH). Electrocardiography (ECG) showed sinus tachycardia with T-wave inversion in lead III (Figure 1). In addition to that, a chest X-ray was done and showed prominent bilateral hilar bronchovascular markings (Figure 2). A CT pulmonary angiogram confirmed the diagnosis of bilateral pulmonary embolism (Figures 3, 4, 5). However, ultrasound Doppler of the lower limbs was unremarkable.

**Table 1:** Investigations upon diagnosis compared to day four of treatment FBC: full blood count; WBC: white blood cells; CK-MB: creatine kinase-myocardial band; NT-proBNP: N-terminal pro-B-type natriuretic peptide; APTT: activated partial thromboplastin time; INR: international normalized ratio

Investigation		Reference range	Units	At diagnosis	Day 4 on LMWH
FBC	WBC	3.6 - 11.0	10^3/uL	18.3*	12.8
	Hemoglobin	12.0 - 15.0	g/dL	9.9*	9.5
	Platelets	150 - 410	10^3/uL	197	246
Inflammatory markers	Procalcitonin (PCT)	< 0.05	ng/mL	0.10*	0.47
	C-reactive protein (CRP)	< 5.0	mg/L	39.3*	45.4
Cardiac enzymes	Troponin T	< 14	ng/L	149	35
	CK-MB	< 4.89	ng/mL	3.4	-
	NT-proBNP	< 125	pg/mL	66.4	228
Coagulation profile	Dimer test	< 0.5	ug/ml FEU	>20.00	0.62
	Prothrombin time	12.2 - 14.6	secs	14.8	12.7
	APTT	28.6 - 38.2	secs	48	45.3
	INR	0.8 - 1.1		1.14	0.95
Urea electrolytes	Sodium	136 - 145	mmol/L	135	139
	Potassium	3.3 - 4.8	mmol/L	3.6	3.9
	Chloride	98 - 108	mmol/L	105	1.7
	Bicarbonate (HCO3)	20 - 28	mmol/L	19.7	19.5
	Urea	12 - 40	mg/dL	11	29
Thrombophilia screening	Factor II	Negative	-	-	Negative
	Factor V	Negative	-	-	Negative

The patient remained hemodynamically stable and was discharged home in stable condition on day five post-delivery. She was kept on a therapeutic dose of enoxaparin for three months, followed by bridging to warfarin till the target international normalized ratio (INR) level was achieved. 

## Discussion

The risk of pulmonary embolism (PE) increases sixfold during pregnancy compared to non-pregnant women [8]. Studies indicate that PE is more prevalent in the puerperium period than deep vein thrombosis (DVT) [9]. Despite the use of prophylactic anticoagulation, some pregnant patients still develop thromboembolic events. This can be attributed to the hypercoagulable state induced by pregnancy, which significantly increases the risk of VTE [10]. The failure of prophylaxis in some cases underscores the need for effective risk assessment tools and individualized patient management strategies. 

Various guidelines and risk assessment tools have been developed to identify pregnant women at risk for VTE and to guide prophylactic measures. The Royal College of Obstetricians and Gynecologists (RCOG), the American College of Obstetricians and Gynecologists (ACOG), and the Society of Obstetricians and Gynecologists of Canada (SOGC) have all established protocols for VTE risk assessment in pregnancy.

The RCOG guidelines recommend a comprehensive risk assessment at the first antenatal visit, during any hospital admission, and postpartum. The RCOG tool stratifies patients into different risk categories based on factors such as previous VTE, thrombophilia, age, obesity, and immobility. Prophylactic low molecular weight heparin (LMWH) is recommended for those identified as high risk from the start of pregnancy, and those who fall in the intermediate risk group should start LMWH from 28 weeks onwards [11].

ACOG emphasizes the importance of individualized risk assessment and recommends thromboprophylaxis for women with a history of VTE, thrombophilia, or other significant risk factors. The ACOG guidelines also highlight the need for postpartum risk assessment and appropriate prophylaxis [12].

The SOGC guidelines provide a detailed approach to VTE risk assessment, incorporating both antepartum and postpartum periods. The SOGC tool includes factors such as previous VTE, family history, and pregnancy-related complications. Prophylactic anticoagulation is advised for women at high risk, with adjustments based on individual patient factors [13].

The efficacy of these risk assessment tools in pregnancy has been a subject of research and debate. Studies have shown that while these tools are effective in identifying high-risk patients and guiding prophylactic measures, there are limitations. For instance, the RCOG tool has been found to be effective in reducing VTE incidence when used consistently, but its predictive accuracy can vary based on population characteristics [14]. Similarly, the ACOG and SOGC tools are valuable in clinical practice but may require adaptation to specific patient populations to enhance their predictive value [10].

Overall, the use of these risk assessment tools has contributed to a reduction in VTE-related maternal mortality [7]. However, continuous evaluation and refinement of these tools are necessary to address their limitations and improve their efficacy in diverse populations.

In the reported case, the patient exhibited several risk factors for venous thromboembolism, including parity, prolonged hospital stays, complete bed rest, immobility, and preterm delivery. Mobility in the context of VTE prophylaxis refers to activities that enhance blood circulation and reduce the risk of clot formation. Clinical guidelines recommend that pregnant women, especially those at higher risk for VTE, should be encouraged to stay mobile. This can include simple activities like walking, leg exercises and avoiding prolonged periods of immobility. In some cases, mechanical prophylaxis such as compression stockings may be used in conjunction with mobility to further reduce the risk. Regular movement helps prevent venous stasis, which is a major contributing factor to thrombus formation [11].

Diagnosing PE can be challenging due to vague symptoms such as shortness of breath, palpitations, and chest pain, which are common in other conditions [9]. Failure to investigate these symptoms is a consistent finding in maternal death inquiries [7]. In this case, the patient developed acute chest pain and shortness of breath with desaturation shortly after delivery and was diagnosed with pulmonary embolism despite being on a prophylactic dose of low molecular weight heparin. 

Prompt initiation of a therapeutic dose of low molecular weight heparin, based on high clinical suspicion of PE, averted lethal consequences. Diagnosis typically involves imaging studies such as CT pulmonary angiography or a ventilation-perfusion scan [11]. Treatment usually involves low molecular weight heparin for at least six months and continuing for a minimum of six weeks postpartum [5]. In our case, the patient received a therapeutic dose of enoxaparin for three months postpartum, followed by warfarin until the target INR was achieved.

Massive PE can cause death within hours due to disseminated intravascular coagulopathy (DIC) and multiorgan failure [15,16]. Therefore, a high index of suspicion, accurate diagnostic approaches, and timely prophylaxis and therapy are crucial for preventing maternal mortality.

## Conclusions

In conclusion, pulmonary embolism (PE) represents a significant risk during pregnancy, necessitating heightened awareness and vigilance among healthcare providers. The complexities of physiological changes during pregnancy, coupled with the potential for increased thrombotic events, underscore the need for effective risk assessment tools and stratification methods. By identifying high-risk individuals and implementing targeted prophylactic measures, clinicians can significantly reduce the incidence of PE and its associated morbidity and mortality. Ultimately, a proactive approach to managing the risk of PE in pregnant patients is vital for ensuring maternal and fetal safety.
